# Circular RNA-Associated Competing Endogenous RNA Network and Prognostic Nomogram for Patients With Colorectal Cancer

**DOI:** 10.3389/fonc.2019.01181

**Published:** 2019-11-08

**Authors:** Wei Song, Tao Fu

**Affiliations:** Department of Gastrointestinal Surgery II, Renmin Hospital of Wuhan University, Wuhan, China

**Keywords:** colorectal cancer, competitive endogenous RNA, circRNA, nomogram, overall survival

## Abstract

**Background:** Genetic characteristics remain underutilized for establishing prognostic models for colorectal cancer (CRC). We explored the underlying regulatory mechanisms of circular RNAs (circRNAs) that act as competing endogenous RNAs (ceRNAs) and constructed a gene-based nomogram to predict overall survival (OS) in patients with CRC.

**Methods:** We obtained circRNA expression profiling data from the Gene Expression Omnibus (GEO) database. MicroRNA (miRNA) and mRNA expression profiles, with associated clinical data, were obtained from The Cancer Genome Atlas (TCGA). A ceRNA network was established using Cytoscape. Interactions between differential genes were analyzed, and hub genes were identified using the cytoHubba application. The R package “clusterProfiler” was used to evaluate the Gene Ontology (GO) annotations of the differentially expressed mRNAs and the Kyoto Encyclopedia of Genes and Genomes (KEGG) pathways. Database-extracted patients were randomized into a training and validation cohorts. A prognostic model was developed using the training set based on multivariate Cox analyses and was then assessed in the validation set. The accuracy of the model was evaluated using discrimination and calibration plots.

**Results:** Thirteen circRNAs, 62 miRNAs, and 301 mRNAs were used to construct the ceRNA network; 10 hub genes were identified via the PPI network. Next, a circRNA- miRNA hub of gene-regulatory modules was established based on four differentially expressed circRNAs, eight differentially expressed miRNAs, and nine differentially expressed mRNAs (DEmRNAs). GO and KEGG pathway analyses indicated the possible association of DEmRNAs with CRC onset and progression. Multivariate analyses revealed that age, tumor stage, and *CXCR5* expression were independent risk factors for OS. A *CXCR5*-based model was developed to predict the OS of patients with CRC in our training set. Our nomogram showed relatively good accuracy, with C-indices of 0.757 and 0.702 in the training and validation sets, respectively. The areas under the curve of the nomograms predicting 3- and 5-years OS were 0.749 and 0.805 in the training set and 0.706 and 0.779 in the validation set, respectively.

**Conclusions:** Our data suggested that the hsa_circ_00001666/has-mir-1229/*CXCR5* axis plays an important role in the pathogenesis of CRC, thereby identifying a potential therapeutic target. The proposed *CXCR5*-based nomogram may also assist surgeons in devising personalized treatments for patients with this disease.

## Introduction

Colorectal cancer (CRC) remains an important global health concern; 1.4 million new cases are diagnosed and 700,000 individuals die of this disease annually. Over the last half-century, the incidence and mortality rates of CRC have been rising yearly (1). Despite improved early detection methods in recent years and the longer survival rates achieved with curative surgery and adjuvant chemotherapy/radiotherapy, CRC remains deadly because of high rates of recurrence and distant metastasis (2, 3). The overall prognosis of CRC remains poor, with 5-years survival rates estimated at 50% (4). Therefore, identifying the key molecular mechanisms of CRC and establishing effective prognostic nomograms for individualized treatment are essential.

Linear non-coding RNAs and circular non-coding RNAs (circRNAs) have been found to be crucial molecules that regulate tumorigenesis and progression (5, 6). CircRNA, a recently discovered class of non-coding RNAs that can regulate eukaryote gene expression (7), are closed RNA molecules that are generated by the selective cleavage of premature mRNA. They are produced by linking the 5′ and 3′ ends generated by backsplicing linear RNA, which makes them resistant to exonucleases and more stable than linear RNAs (8).

Salmena et al. (9) first presented their hypothesis of the existence of competitive endogenous RNA (ceRNA) in 2011. The hypothesis suggests that circRNAs can act as natural miRNA sponges that restrain their function because they share miRNA response elements. Accumulating evidence suggests that circRNAs play a role in many pathophysiological and physiological processes. For example, circRNA circ_0052112 is an miR-125a-5p sponge that regulates cellular migration and invasion during the development of breast cancer (10). Furthermore, circRNA_NEK6 can enhance thyroid cancer cell proliferation by competitively binding miR-370-3p and upregulating FZD8 (11).

Presently, the American Joint Committee on Cancer's tumor-node-metastasis (TNM) staging system is essential for selecting optimal clinical interventions (12). While this system is effective for patient populations, it is not very useful for predicting individual patient outcomes. Moreover, there are several factors, such as sex, age, tumor site, and molecular biomarkers, that can influence the patient's survival. Therefore, developing more accurate CRC outcome prediction models is warranted.

Nomograms are statistics-based tools that are used to calculate the risk of clinicopathological cancer features and have been widely developed to assess the survival rate of patients with cancer (13). Several models have been established for predicting the prognosis of CRC using various clinicopathologic characteristics, such as age, sex, tumor size, location, histology, differentiation, and T, N, and M stages (14). Although these studies have produced significant knowledge, few have considered the use of genetic characteristics to establish a prognostic model for CRC. Therefore, the deeper understanding of the genetic characteristics of CRC that is necessary for developing a better model to predict patient prognoses remains a critical unmet need.

In this study, we integrated data from the public Gene Expression Omnibus (GEO) and “The Cancer Genome Atlas” (TCGA) databases to construct a novel circRNA-associated ceRNA network for CRC. We then established an mRNA-based prognostic nomogram of patients with CRC using univariate and multivariate Cox regression analysis. Our resulting nomogram exhibited good discrimination and calibration. Our data further clarify the roles of circRNAs in the pathogenesis of CRC and can serve as a guide toward more effective individualized treatment decisions for patients with this disease.

## Materials and Methods

### Dataset Collection

The circRNA expression profiles (GSE126095) were obtained from the GEO (https://www.ncbi.nlm.nih.gov/geo/) database, including data from 10 CRC and 10 normal tissues. Clinical information from 547 patients with CRC was obtained, as were the expression data of mRNA (571 CRC tissues and 44 normal tissues) and miRNA (542 CRC tissues and 9 normal tissues) from TCGA (https://portal.gdc.cancer.gov/). Sixty-eight CRC patients were excluded because of unknown survival time (36 patients), age (14 patients), and tumor stage (18 patients). Ultimately, 493 patients were included in this study. Approval by the institutional ethics committee was not necessary because all data were collected from the publicly available GEO and TCGA databases.

### Identifying Differentially Expressed Genes

The expression profiles of miRNA and mRNA in “fragments per kilobase of transcript per million mapped reads” for patients with CRC were downloaded from the TCGA database. circRNA expression profile data in raw read counts were downloaded for the GEO database. These raw data were processed by background correction and normalization using the “affy” package of R/Bioconductor. The “limma” package was used to identify the differentially expressed circRNAs (DEcircRNAs), while the edgeR package was used to identify the differentially expressed miRNAs (DEmiRNAs) and mRNAs (DEmRNAs) between normal samples and tumor samples. The cut-off values of circRNA were set at the adjusted *P*-value of < 0.01 and |log2 fold change (FC)| >3.0, while the cut-off values of miRNA and mRNA were set at the adjusted *P*-value of < 0.01 and |log2 FC| >2.0.

### Constructing the ceRNA Network

A circRNA-associated ceRNA network was established based on the relationships between DEcircRNAs, DEmiRNAs, and DEmRNAs, and was visualized with Cytoscape (version 3.6.0; www.cytoscape.org). The circRNA-miRNA interactions were predicted using the Cancer-Specific CircRNA (http://gb.whu.edu.cn/CSCD/) database. To maximize the reliability of the data, these target miRNAs were further screened by the DEmiRNAs obtained from the TCGA database. The miRNA-mRNA interactions were predicted using the TargetScan and miRTarBase databases (15, 16). Only mRNAs recognized by both databases were considered candidate targets and were intersected with the identified DEmRNAs to screen out the DEmRNAs that were targeted by the DEmiRNAs.

### Protein-Protein Interaction (PPI) Network

To further explore the interaction among the DEmRNAs, a PPI network of DEmRNAs was identified using the Search Tool for the Retrieval of Interacting Genes (STRING) (http://string-db.org/) database (17). This database has a comprehensive score for each PPI relationship pair that is distributed between 0 and 1 [25]; the higher the total score, the more reliable the PPI relationship. The commonly used combined score threshold was 0.4 (18, 19). In this study, we used an interaction score >0.7 as the cut-off criterion. To identify crucial circRNA-miRNA- mRNA subnetworks, the hub genes in this PPI network were extracted by using the cytoHubba application (20), while the Cytoscape software (version 3.7.0) was used to visualize the PPI network.

### Functional Enrichment of Differentially Expressed Genes

In order to identify the Gene Ontology (GO) annotations and pathways in which hub genes were enriched, GO term and Kyoto Encyclopedia of Genes and Genomes (KEGG) pathway enrichment analyses were performed using the “clusterProfiler” package in R/Bioconductor (21).

### Variables

The characteristics of patients with CRC (age, sex, race, tumor location, TNM stage, 10 hub genes, and follow-up information) were extracted. The primary endpoint was overall survival (OS), which was defined as the interval between the date of diagnosis and that of the most recent follow-up or of death from any cause. All patients were randomly categorized into the training or validation set with a ratio of 7:3.

### Statistical Analyses

#### Construction of the Nomogram

Categorical data are shown as frequencies and proportions and were compared using the Chi-Squared or Fisher's exact test. Survival curves were created using the Kaplan-Meier method and compared using the log-rank test. We used Cox's proportional hazards regression model for estimating the hazard ratios (HRs) and 95% confidence intervals (95% CIs). Risk factors were identified using forward stepwise selection and Cox proportional hazards regression, based on which we constructed a nomogram for predicting the 3- and 5-years OS in the training set.

#### Nomogram Validation

Both external and internal cohorts (i.e., validation and training sets, respectively) were used to test the generalizability of the nomogram; discrimination and calibration were used to assess its predictive ability and compliance. The discriminatory ability of the nomogram was evaluated by calculating the concordance index (C-index), which is similar to the area under the receiver operating characteristic curve (AUC) and measures the variation between the predicted and observed outcomes (22). Calibration plots were used to compare the observed and predicted probabilities for the nomogram. Data analyses were performed using the software SPSS version 23 (IBM Corp., Armonk, NY, USA), along with version 3.5.1 of the R software (Institute for Statistics and Mathematics, Vienna, Austria; r-project.org). A *P*-value < 0.05 was considered statistically different.

## Results

### Patient Characteristics

A total of 493 patients were included and were randomly assigned into a training set (*n* = 346) for designing the nomograms and a validation set (*n* = 147). In both cohorts, most patients with CRC were elderly (≥65 years) and Caucasian. The most common CRC tumor site for CRC was the colon (84.8%), and most patients had stage II (38.7%) or III (27.6%) disease. The clinicopathological characteristics of the patients are summarized in Table 1.

**Table 1:** Clinicopathologic characteristics of CRC patients included in this study.

**Variables**	**All patients**	**Training set**	**Validation set**
	**(*n* = 493)**	**(*n* = 346)**	**(*n* = 147)**
	***N* (%)**	***N* (%)**	***N* (%)**
Age (M ± SD, years)	65.5 ± 12.6	65.7 ± 12.7	65.1 ± 12.4
**Sex**			
Male	271 (55.0%)	184 (53.2%)	87 (59.2%)
Female	222 (45.0%)	162 (46.8%)	60 (40.8%)
**Race**			
White	230 (46.7%)	156 (45.1%)	74 (50.3%)
Black	62 (12.6%)	45 (13.0%)	17 (11.6%)
Other	201 (40.8%)	145 (41.9%)	56 (38.1%)
**Tumor site**			
Rectum	75 (15.2%)	48 (13.9%)	27 (18.4%)
Colon	418 (84.8%)	298 (86.1%)	120 (81.6%)
**Stage**			
I	84 (17.0%)	59 (17.1%)	25 (17.0%)
II	191 (38.7%)	136 (39.3%)	55 (37.4%)
III	136 (27.6%)	94 (27.2%)	42 (28.6%)
IV	82 (16.6%)	57 (16.5%)	25 (17.0%)

### Identification of Differentially Expressed Genes in CRC

**Figure 1:**
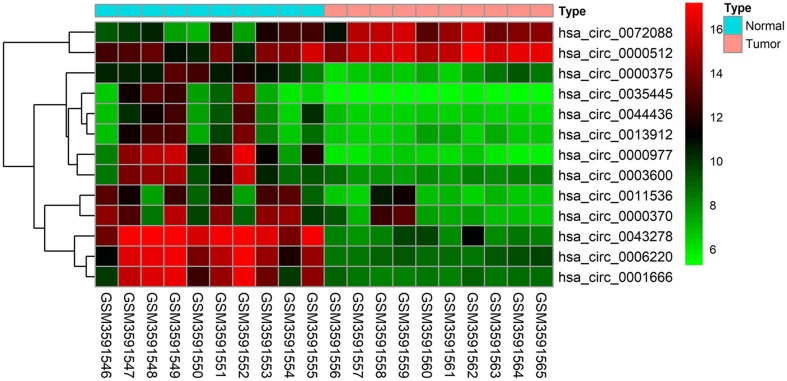
Heat map of the 13 differentially expressed circular RNAs (circRNAs) in the GSE126095 dataset. The X-axis represents the samples, while the Y-axis denotes the differentially expressed circRNAs. Green and red tones represent downregulated and upregulated genes, respectively.

**Figure 2:**
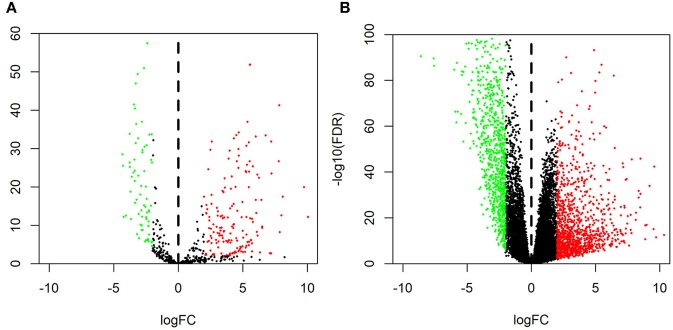
Volcano plot of differentially expressed RNAs. Normalized expression levels are shown in descending order from green to red. Shown are microRNAs **(A)** and mRNAs **(B)** from The Cancer Genome Atlas.

The GSE126095 dataset from the GEO was analyzed using the R project “limma” package (adjusted *P*-value < 0.01 and |log2 FC| >3.0); a total of 13 DEcircRNAs were identified, of which 2 were up-regulated and 11 were down-regulated (Figure 1). The data from TCGA were analyzed using the “edgeR” package in R (adjusted *P*-value < 0.01 and |log2 FC)| >2.0), and 255 differentially expressed miRNAs (including 168 and 87 that were upregulated and downregulated, respectively) as well as 2,071 differentially expressed mRNAs (including 1,158 and 913 that were upregulated and downregulated, respectively) were identified (Figures 2A,B).

### Construction of the ceRNA Network

**Figure 3:**
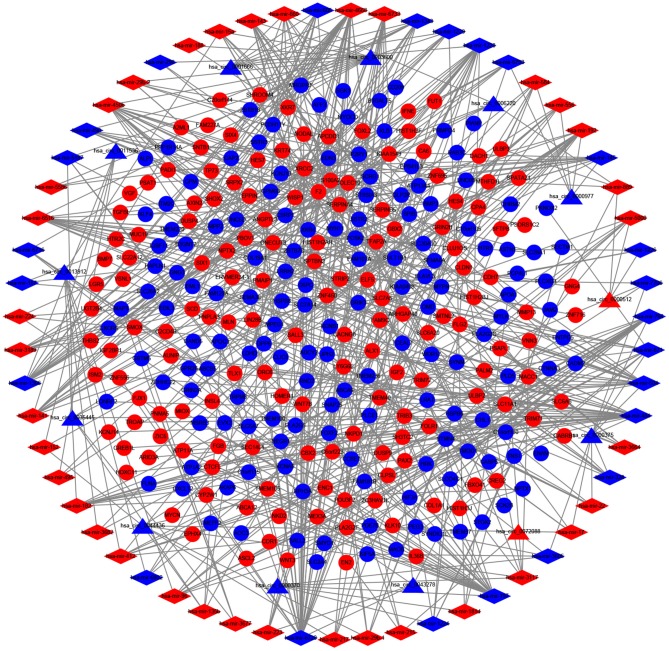
The competing endogenous RNA (ceRNA) network of circular RNA (circRNA)- microRNA (miRNA)-mRNA in colorectal cancer. Triangles represent circRNAs, diamonds represent miRNAs, ellipses represent mRNAs, and gray lines represent circRNA-miRNA-mRNA interactions. Red nodes indicate upregulated expression, whereas green nodes denote downregulated expression.

A circRNA-miRNA-mRNA network was constructed and analyzed using Cytoscape v3.6.0. We used the CSCD online database to identify DEcircRNAs with the target miRNAs; a total of 814 circRNA-miRNA pairs were constructed based on 13 DEcircRNAs and 522 miRNAs. After cross-checking with the DEmiRNAs, only 62 circRNA-miRNA pairs remained. We further identified mRNAs targeted by these DEmiRNAs from the miRTarBase and TargetScan databases and selected those that overlapped with the identified DEmRNAs. Finally, a total of 301 DEmRNAs were included in the ceRNA network, along with 13 circRNAs and 62 miRNAs (Figure 3).

### Construction of the PPI Network

**Figure 4:**
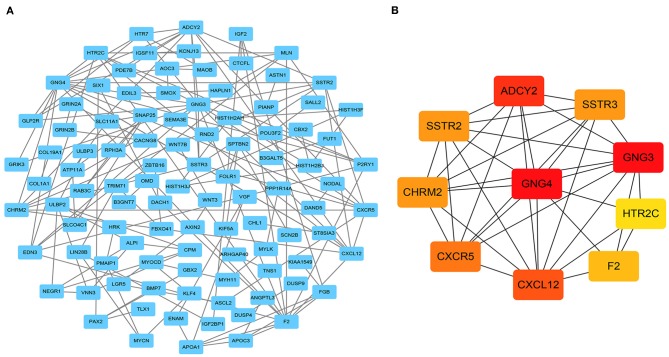
Identification of hub genes from the protein-protein interaction (PPI) network. **(A)** PPI network of 301 genes, consisting of 101 nodes and 146 edges. **(B)** PPI network of 10 hub genes extracted from **(A)**. The node color changes gradually from yellow to red in ascending order according to the log2(foldchange) of the genes.

To further explore the interactions between the 301 DEmRNAs, a PPI network was constructed (Figure 4A). After removing unconnected nodes, the PPI network contained 101 nodes and 146 edges. Furthermore, the hub genes in the PPI network that had high degrees of connectivity were identified using the cytoHubba plugin for Cytoscape. The 10 most significant genes were *GNG4, GNG3, ADCY2, CXCL12, CXCR5, CHRM2, SSTR2, SSTR3, F2*, and *HTR2C* (Figure 4B). Next, a circRNA- miRNA-hub_gene network was constructed, including 4 circRNAs (hsa_circ_0000375, hsa_circ_0001666, hsa_circ_0003600, and hsa_circ_0011536), 9 miRNAs (hsa-mir-22, hsa-mir-328, hsa-mir-504, hsa-mir-1228, hsa-mir-1229, hsa- mir-4668, hsa-mir-29b-2, hsa-mir-6516, and hsa-mir-1306), and 10 hub genes.

### Functional Enrichment Analysis of DEmRNAs

**Figure 5:**
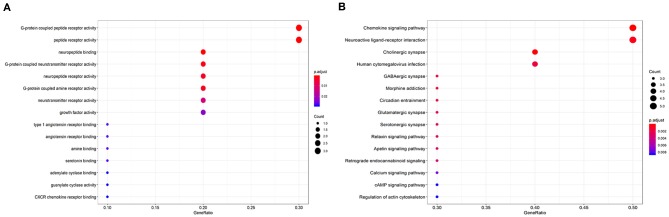
Enrichment of the top 15 Gene Ontology terms **(A)** and Kyoto Encyclopedia of Genes and Genomes pathways **(B)** of differentially expressed mRNAs in the competing endogenous RNA network. The node color changes gradually from red to blue in ascending order according to the adjusted *P*-values. The size of each node represents the number of counts.

GO enrichment and KEGG pathways were analyzed to investigate the functions of the hub genes. A total of 32 GO terms together with 23 KEGG pathways were significantly enriched; the significant GO terms were G-protein-coupled peptide receptor activity (*P* = 0.001) and peptide receptor activity (*P* = 0.001). Moreover, the top 10 KEGG analysis pathways included “Chemokine signaling pathway,” “Cholinergic synapse,” “Neuroactive ligand-receptor interaction,” “Human cytomegalovirus infection,” “GABAergic synapse,” “Morphine addiction,” “Circadian entrainment,” “Glutamatergic synapse,” “Serotonergic synapse,” and “Relaxin signaling pathway.” The top 15 GO terms and KEGG analysis pathways are shown in Figures 5A,B.

### Development and Validation of the Nomogram Based on *CXCR5*

**Figure 6:**
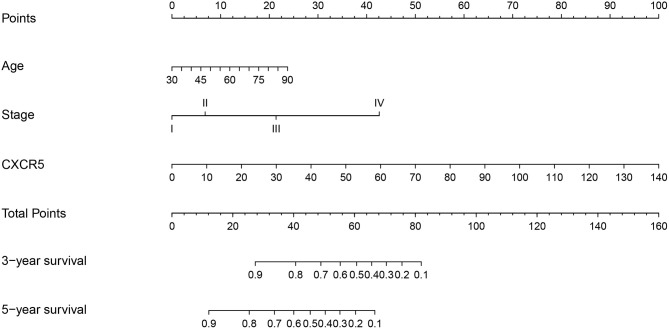
A nomogram for the prediction of 3- and 5-years overall survival in patients with colorectal cancer.

**Figure 7:**
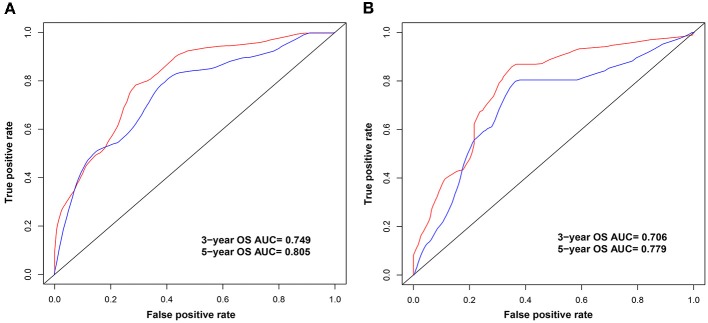
Area under the curves (AUCs) of the nomogram for the prediction of 3- and 5-years overall survival in the training **(A)** and validation **(B)** sets. The blue and red lines represent the nomogram-predicted 3- and 5-years overall survival rates, respectively.

**Figure 8:**
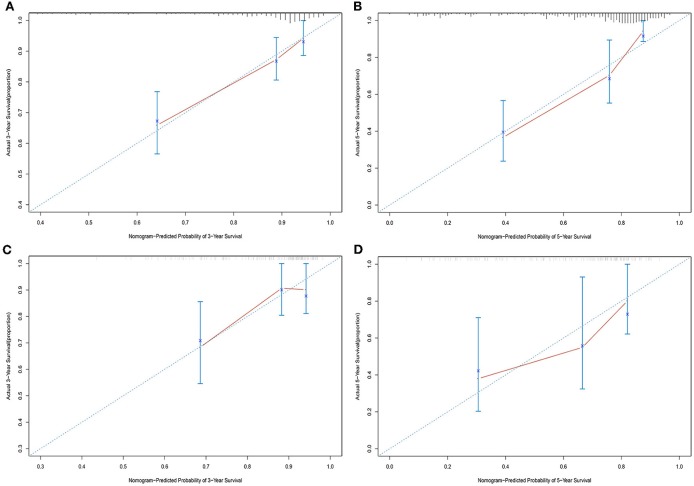
Calibration plots of the nomogram for 3- and 5-years overall survival predictions in the training set **(A,B)** and validation set **(C,D)**.

Univariate analysis of OS in patients with CRC using Cox regression analysis demonstrated that age, stage, and expression of *SSTR2, CXCR5*, and *SSTR3* significantly influenced OS in patients within the training set (Table 2). Multivariate analysis indicated that three variables (age, stage, and *CXCR5* expression) were independent risk factors for OS. A nomogram predicting 3- and 5-years OS was constructed based on the multivariate analysis data (Figure 6). The points from each independent prognostic factor listed in the nomogram were summed. The nomograms were both internally and externally validated; internal validation using the training set revealed a nomogram C-index of 0.757 (95% CI = 0.690–0.824). On external validation, the C-index of the nomogram was 0.702 (95% CI, 0.584–0.820) in terms of predicting OS. Furthermore, the reliability of the nomogram was analyzed using the AUC values. For the training set, the AUC values of the ability of the nomogram to predict the 3- and 5-years OS rates were 0.749 and 0.805, respectively, whereas the corresponding values in the validation set were 0.706 and 0.779, respectively (Figure 7). The calibration plots indicated no apparent departure from the ideal line, with optimal agreement achieved between the nomogram-predicted outcomes and actual survival rates in both the training and validation sets (Figure 8).

**Table 2:** Univariate and multivariate analyses of overall survival in the training set.

**Variable**	**Univariate analysis**		**Multivariate analysis**	
	**HR (95%CI)**	***P*-value**	**HR (95%CI)**	***P*-value**
Age	1.026 (1.0033–1.051)	0.026	1.022 (1.000–1.045)	0.037
**Sex**				
Male	1		1	0.883
Female	0.988 (0.606–1.609)	0.960	1.041 (0.609–1.780)	
**Race**				
White	1		1	
Black	1.272 (0.643–2.519)	0.490	1.005 (0.504–2.004)	0.989
Other	0.819 (0.469–1.433)	0.486	0.977 (0.554–1.724)	0.936
**Tumor site**				
Rectum	1		1	
Colon	2.144 (0.779–5.903)	0.140	2.561 (0.916–7.161)	0.073
**Stage**				
I	1		1	
II	1.510 (0.433–5.271)	0.518	1.514 (0.433–5.293)	0.516
III	3.263 (0.964–11.043)	0.057	3.209 (0.941–10.947)	0.063
IV	10.080 (3.066–33.136)	<0.001	10.517 (3.181–34.773)	<0.001
GNG3	1.014 (0.987–1.046)	0.391	1.015 (0.976–1.055)	0.462
HTR2C	1.005 (0.997–1.013)	0.225	1.004 (0.995–1.0133)	0.396
F2	0.997 (0.990–1.004)	0.370	0.996 (0.988–1.003)	0.273
SSTR2	1.004 (1.002–1.006)	<0.001	1.000 (0.992–1.008)	0.969
CXCR5	1.039 (1.022–1.056)	<0.001	1.047 (1.003–1.093)	0.034
CHRM2	0.998 (0.995–1.002)	0.326	0.996 (0.992–1.000)	0.092
CXCL12	1.000 (0.999–1.000)	0.827	1.000 (0.999–1.000)	0.659
SSTR3	1.022 (1.012–1.032)	<0.001	0.996 (0.947–1.048)	0.886
GNG4	1.000 (0.999–1.000)	0.805	1.000 (0.999–1.000)	0.378
ADCY2	1.002 (0.1000–1.003)	0.058	1.001 (0.999–1.002)	0.128

## Discussion

We identified genes that are dysregulated in CRC based on publicly available databases (GEO and TCGA). Based on the joint analysis of multiple databases, a CRC-centric circRNA-miRNA-mRNA ceRNA network was established. We also established a PPI network to highlight hub genes that may play critical roles in the pathogenesis of CRC. Moreover, a circRNA-miRNA-hub_gene network was also constructed, and we performed functional analyses to determine the biological roles of hub genes in CRC. Finally, we established and validated nomograms to predict 3- and 5-years OS in patients with CRC based on *CXCR5* gene expression. The model exhibited good predictive capability in both the training and validation sets. Our data provide novel insights into the circRNA-related ceRNA network in CRC and also identified potential therapeutic targets.

circRNAs are a unique class of RNAs formed by the splicing of RNA loops (23) and are diverse, stable, and conserved molecules (24, 25). Based on these properties, circRNAs are potential cancer biomarkers (26, 27). Recent studies have shown that circRNAs are dysregulated in various types of cancer, including CRC, and may be associated with cancer progression as well as patient survival. For example, Chi et al. (28) found that hsa_circ_0000285 was lower in bladder cancer tissues and serum than in adjacent normal tissues and that patients with reduced hsa_circ_0000285 exhibited advanced clinicopathological features and worse OS. As such, they considered this circRNA to be a prognostic biomarker for patients with bladder cancer. Similarly, circ-CBFB was found to be highly expressed in chronic lymphocytic leukemia cells; its knockdown significantly inhibited the proliferation of these cells, prevented cell cycle progression, and induced apoptosis. Further studies revealed that circ-CBFB acts as a sponge for miR-607 to reduce its levels, promote expression of the *FZD3* target gene, and promote the activation of the Wnt/β-catenin pathway and subsequent progression of chronic lymphocytic leukemia (29). However, the exact role of circRNA-related ceRNA networks in CRC development remains largely elusive. In our study, we identified 4 DEcircRNAs in the circRNA-miRNA- hub_gene network and found that hsa_circ_0001666 was downregulated in CRC tissues. This result is consistent with a recent study (30) indicating that hsa_circ_0001666 was downregulated in breast cancer tissues and may therefore play a key role in the development of breast malignancies. However, other circRNAs discovered in our ceRNA network have not been reported previously. Therefore, the roles of these circRNAs in CRC require further validation in future studies.

miRNAs are a type of endogenous non-coding RNA that are ~19–25 nucleotides long and have widespread physiological activity. They influence the development, invasion, metastasis, and prognosis of various types of cancer by regulating the expression levels of oncogenes and tumor suppressor genes. Various miRNAs have been found to be associated with the development and progression of CRC, such as miR-218, miR-497, and miR-101 (31–33). Upregulation of miR-218 significantly inhibits epithelial-to-mesenchymal transition and angiogenesis in CRC cells by targeting connective tissue growth factor (31). Furthermore, the overexpression of miR-497 inhibits the proliferation, migration, and invasion of multiple myeloma cells through the MAPK/ERK signaling pathway by targeting Raf-1 (32). In the present study, we identified nine miRNAs involved in the circRNA- miRNA-hub_gene sub-network, of which miR-22, miR-328, miR-1229, and mir-29b-2 have previously been described as having roles in CRC (34–37). For example, miRNA-22 inhibits the growth, migration, and invasion of CRC cells through a Sp1-mediated negative feedback loop (35). Overexpression of miR-1229 in exosomes extracted from the serum of patients with CRC promotes angiogenesis by targeting homeodomain-interacting protein kinase-2 and is significantly correlated with advanced TNM stage, lymphatic metastasis, and poor prognosis (36).

We constructed a PPI network to investigate the functional interactions between the DEmRNAs we identified and selected the 10 hub genes with the highest degree of connectivity. Some of these genes have been reported to be closely associated with CRC, such as *CXCL12, CXCR5, SSTR2*, and *F2*. Yu et al. (38) found that *CXCR12* promotes inflammatory CRC progression by recruiting immune cells and enhances cytoskeletal remodeling via signaling through the linear non-coding RNA XIST/miR-133a-3p/RhoA. Yan et al. (39) found that high tumor *CXCR5* expression (compared to adjacent normal tissue) can promote the pathogenesis, metastasis, and recurrence of CRC, suggesting that this gene is a valuable predictor of prognosis in patients with CRC.

Functional annotation and pathway enrichment analysis using the 10 identified hub genes provided an intuitive overview of the mechanism of CRC. The significant GO terms were G-protein coupled peptide receptors and other peptide receptors that are involved in inflammatory immune responses, as are chemokine receptors. Moreover, KEGG pathway enrichment analysis showed significant enrichment of chemokine signaling pathways, including of the genes *GNG4, GNG3, ADCY2, CXCL12*, and *CXCR5*. The CXC family of chemokines and their receptors are crucial for modulating inflammation and antitumor immunity, both of which are key factors in CRC progression. These chemokines modulate tumor behavior by promoting angiogenesis, activating tumor-specific immune responses, and directly stimulating tumor proliferation in an autocrine or paracrine manner. The *CXCR5* axis is generally considered to have tumor suppressor properties, and its stimulation has been proposed as a means of combating cancer (40). Taken together, these findings indicate that CXCR5 may be involved in the pathogenesis, metastasis, and recurrence of CRC. Therefore, based on the circRNA- miRNA-hub_gene network, the hsa_circ_00001666/has-mir-1229/CXCR5 axis may play an important role in the development of CRC.

To establish a relatively accurate prognostic model for patients with CRC, we performed univariate and multivariate Cox analyses of various factors including age, sex, race, tumor location, TNM stage, and the 10 identified hub genes. The result showed that age, stage, and CXCR5 were independent risk factors of OS for patients with CRC. Our novel prognostic nomogram, which included both mRNA expression and clinical information, exhibited strong discriminatory abilities, was well-calibrated, and showed good prediction of OS. Therefore, our proposed nomogram has promising prospects so far as clinical application is concerned.

Several limitations of our study should be noted. First, the ceRNA network we constructed was based on bioinformatics analysis; the circRNA-miRNA-mRNA regulatory network needs to be validated both *in vitro* and *in vivo*. Second, owing to the lack of relevant prognostic data, we were unable to evaluate the prognostic value of DEcircRNAs in patients with CRC, and these were therefore not incorporated into the nomogram. Third, the training and validation sets were from the same database; external clinical data from independent sources are needed to validate our nomogram properly. Lastly, our sample size was relatively small.

## Conclusions

We identified a ceRNA network that describes the possible mechanisms of CRC and can shed light on the heretofore unknown regulatory network(s) of ceRNAs in CRC. Multivariate analysis showed that *CXCR5* is an independent prognostic factor, suggesting that the hsa_circ_00001666/has-mir-1229/*CXCR5* axis may play an important role in the development of CRC. Moreover, the prognostic nomogram that we established based on *CRCR5* displayed favorable discrimination and good prediction of prognosis. Our data indicate that the hsa_circ_00001666/has-mir-1229/*CXCR5* axis plays an important role in the pathogenesis of CRC and therefore constitutes a potential therapeutic target for patients with this disease. Our proposed nomogram may also help surgeons perform personalized survival evaluations in their patients.

## Data Availability Statement

The raw data supporting the conclusions of this manuscript will be made available by the authors, without undue reservation, to any qualified researcher.

## Ethics Statement

Ethical approval was not provided for this study on human participants because approval by Ethics Committee would not be necessary because all data had been collected from public availability of data in the GEO and TCGA databases. Written informed consent for participation was not provided by the participants' legal guardians/next of kin. Written informed consent was obtained from the individual(s), and minor(s)' legal guardian/next of kin, for the publication of any potentially identifiable images or data included in this article.

## Author Contributions

WS and TF designed the manuscript, performed the experiments, analyzed and interpreted the data, and wrote the manuscript. The final manuscript was read and approved by all authors.

### Conflict of Interest

The authors declare that the research was conducted in the absence of any commercial or financial relationships that could be construed as a potential conflict of interest.
